# Heavy Metal Accumulation by Periphyton Is Related to Eutrophication in the Hai River Basin, Northern China

**DOI:** 10.1371/journal.pone.0086458

**Published:** 2014-01-22

**Authors:** Wenzhong Tang, Jingguo Cui, Baoqing Shan, Chao Wang, Wenqiang Zhang

**Affiliations:** 1 State Key Laboratory on Environmental Aquatic Chemistry, Research Center for Eco-Environmental Sciences, Chinese Academy of Sciences, Beijing, China; 2 Beijing Sound Environmental Engineering Co., Ltd., Beijing, China; CINVESTAV-IPN, Mexico

## Abstract

The Hai River Basin (HRB) is one of the most polluted river basins in China. The basin suffers from various types of pollutants including heavy metals and nutrients due to a high population density and rapid economic development in this area. We assessed the relationship between heavy metal accumulation by periphyton playing an important role in fluvial food webs and eutrophication in the HRB. The concentrations of the unicellular diatoms (type A), filamentous algae with diatoms (type B), and filamentous algae (type C) varied along the river, with type A dominating upstream, and types B then C increasing in concentration further downstream, and this was consistent with changes in the trophic status of the river. The mean heavy metal concentrations in the type A, B and C organisms were Cr: 18, 18 and 24 mg/kg, respectively, Ni: 9.2, 10 and 12 mg/kg, respectively, Cu: 8.4, 19 and 29 mg/kg, respectively, and Pb: 11, 9.8 and 7.1 mg/kg respectively. The bioconcentration factors showed that the abilities of the organisms to accumulate Cr, Ni and Pb decreased in the order type A, type B, then type C, but their abilities to accumulate Cu increased in that order. The Ni concentration was a good predictor of Cr, Cu and Pb accumulation by all three periphyton types. Our study shows that heavy metal accumulation by periphyton is associated with eutrophication in the rivers in the HRB.

## Introduction

Heavy metal pollution in aquatic ecosystems has been a serious global environmental problem for a long time [1], [2], [3]. Heavy metals are persistent in aquatic environments because of their resistance to decomposition under natural conditions [4], [5]. One of the greatest problems associated with the persistence of heavy metals is the potential for them to bioaccumulate and biomagnify, potentially resulting in long-term implications for human and aquatic ecosystem health [6], [7], [8], [9]. Periphyton is an important aquatic resource and is a significant component in river ecosystems [10], [11], it plays an important role in fluvial food webs [12], [13]. Periphyton is highly sensitive to environmental stressors [14], [15], and is often used as a pollution indicator to assess water quality [13], [16], [17]. Heavy metal accumulation by periphyton has a strong effect on river ecosystems because of these factors.

The Hai River Basin (HRB) is one of the most polluted river basins in China. It is in an area that has a high population density and that is undergoing rapid economic development. The HRB suffers from various types of pollution [18]. Heavy industrial development and rapid urbanization have caused significant pollution of the rivers in the HRB, and the main pollutants include nitrogen (N), phosphorus (P) and heavy metals. Many rivers in this area have also degenerated into shallow streams because of excessive water extraction, and this has caused periphyton (mainly benthic algae) to become the primary producer. The ecological degradation of a river can cause enormous changes in the periphyton community, and the periphyton species composition is routinely used as an indicator of heavy metal pollution in a river [19], because periphyton can accumulate metals from the ambient water and from sediments [20]. Therefore, it is important to understand the relationship between heavy metal accumulation by periphyton and various other pollutants (including N and P) in the HRB, in order to provide a reference point for the future control and management of heavy metals in the river ecosystem.

Periphyton is an important food source for invertebrates and some fish, and can be an important accumulator of heavy metals [21]. These accumulated metals may be transferred from periphyton to the consuming organisms [22]. We studied heavy metal accumulation by periphyton in the HRB rivers, and our specific objectives were to: 1) assess the effects of eutrophication (i.e., increased N and P concentrations) on periphyton community succession; 2) determine the degree of heavy metal accumulation in different periphyton community types; 3) assess the effects of eutrophication on heavy metal accumulation by periphyton.

## Materials and Methods

### Ethics Statement

No specific permits were required for the field studies described here. The study area is not privately-owned or protected in any way, and the field studies did not involve endangered or protected species.

### Study Area

The HRB is mainly within Hebei Province, and includes Beijing, Tianjin and parts of Inner Mongolia, Shanxi, Henan and Shandong provinces. The HRB has an area of 318,000 km^2^ and a temperate continental monsoon climate. The mean annual precipitation is 527 mm. The HRB is one of several major river basins managed by the Chinese Ministry of Water Resources. Heavy industrial development and rapid urbanization have caused significant pollution in the aquatic environments in this region. Water resources are in high demand, and deteriorating water quality has exacerbated the shortage of water resources. The HRB has, therefore, attracted much attention from the Chinese government and it has become one of the most important basins in the national 11^th^ and 12^th^ five-year plans for water pollution control.

**Figure 1:**
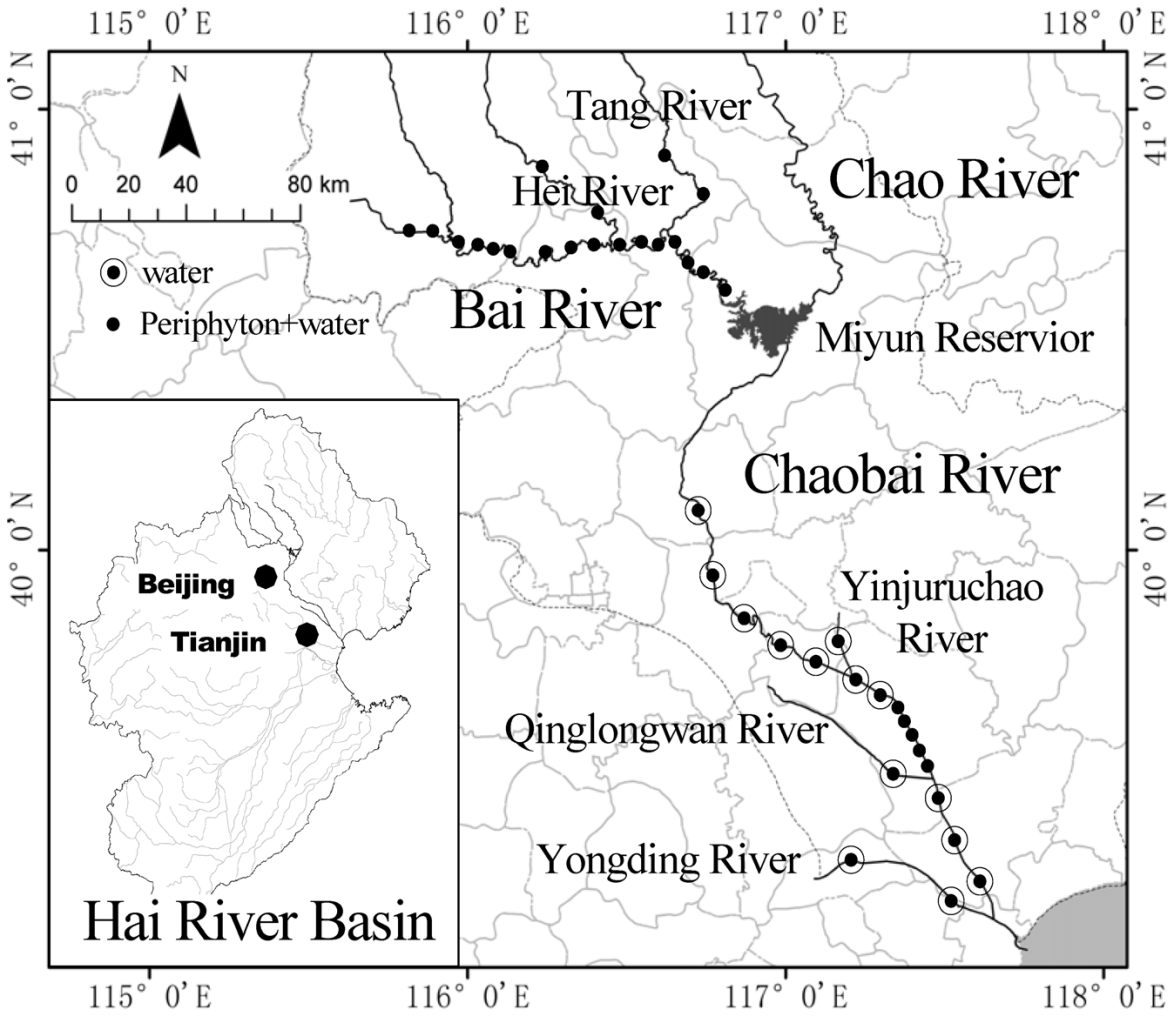
Location of the Chaobai River Watershed in the Hai River Basin and the distribution of the sampling sites.

Chaobai River Watershed (CRW), which is one of the most important watersheds in the HRB, was selected as the study area (Fig. 1). This watershed is a significant source of drinking water for Beijing. Miyun reservoir, which is 100 km northeast of Beijing, feeds the Chao and Bai rivers. Drought and water overuse in recent years have caused some downstream parts of the Chao River to have a decreased flow, and the Bai River has turned into a subalpine shallow stream with a gravel and cobble riverbed. Arable fields lie on either side of the Bai River, and the runoff from these fields produces agricultural non-point-source pollution. The Bai and Chaobai rivers and their tributaries (the Hei, Tang, Yinjuruchao, Qinglongwan and Yongding rivers) in the CRW were selected as the main study sites because the water quality in these systems covers all of the trophic classes (oligotrophic, mesotrophic, eutrophic and hypereutrophic), allowing us to study periphyton responses. Water and periphyton samples were collected from 38 study sites in May and June.

### Chemical Analysis of Water Samples

Water samples (three replicates) were acidified to below pH 2 and analyzed in the laboratory within 72 h of collection. A potassium persulfate digestion was used to prepare the samples before the total N (TN) and total P (TP) concentrations were determined [23]. The TN concentrations were determined using an automated chemical analysis instrument (Smart Chem 200, Westco, Italy), and the detection limit was 0.001 mg/L. Samples for heavy metal analysis were collected in clean acid-washed glass bottles, acidified with concentrated HNO_3_, stored at 4°C, and analyzed within 72 h of collection. TP and heavy metals were all analyzed by inductively coupled plasma-mass spectrometry (ICP-MS) (7500a, Agilent, USA), which had a detection limit 0.001 µg/L for each analyte.

### Periphyton Sampling and Elemental Analysis

Our field investigations revealed great differences in the periphyton community structures present in the HRB, from small epilithic/epiphytic diatoms to large filamentous algae. Sampling sites with similar conditions were selected to avoid physical and hydrological parameters (such as light, shade, substrata, water depth and flow velocity) affecting the results. Periphyton was collected from rock surfaces at each site using a nylon-bristled brush. If there was enough periphyton available, at least three samples were collected at each site each month. Each sample was divided into three aliquots for analysis. The first aliquot was preserved in 3–5% glutaraldehyde solution in the field [24], and the community structure was identified using a microscope (Olympus BX51, Japan) in the laboratory. The second aliquot was frozen in the field, stored at −16°C in a cooler (Mobicool, BC55 DC, USA), freeze dried in the laboratory, then ground into a powder and passed through a 100 mesh sieve. The sample powder was then digested using a microwave digestion system (CEM, Matthews, NC, USA) and analyzed for heavy metals (Cr, Cu, Ni and Pb) by ICP-MS (7500a, Agilent, USA). The recoveries varied but were all within the range 90–95%, and the precision was good, with a relative standard deviation (RSD) of less than 3%. The last aliquot was stored for use as a backup in case the analysis of either of the other aliquots failed.

### Bioconcentration Factor

The bioconcentration factor (BCF) was used to assess the accumulation efficiency of the heavy metals, by comparing the concentration in the biota with the concentration in the external medium [25]. We calculated the BCF using Eq. 1
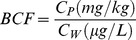
(1)where C_P_ and C_W_ are the heavy metal concentrations in the biota (mg/kg) and the water (µg/L), respectively. A BCF greater than 1 indicates that the periphyton enriched heavy metals from the water.

### Statistical Analysis

The experimental data were analyzed using SPSS 17.0 for Windows. Spearman correlation was used to assess the relationships between the heavy metal concentrations in the three periphyton types. A one-sample t-test (p≤0.05) was used to analyze the variance. Origin Pro 8.0 was used to plot the experimental data.

## Results and Discussion

### Chemical Characteristics of the River Water

Table 1 shows the TN, TP and heavy metal concentrations in the water from the rivers in the CRW that were studied. Overall, the TN and TP concentrations clearly increased moving from the upstream part of the study area (the Bai River and its tributaries) to the downstream part (the Chaobai River and its tributaries). The Cr, Ni and Cu concentrations were about six times higher in the upstream than in the downstream river water, but the Pb concentrations were almost the same. Defining the trophic state is more difficult in rivers than in lakes [26], but the US Environmental Protection Agency has suggested boundaries for the trophic classification of rivers [27], and these are that the oligotrophic–mesotrophic boundary is at TN = 0.70 mg/L and TP = 25 µg/L and that the mesotrophic–eutrophic boundary is at TN = 1.5 mg/L and TP = 75 µg/L. Using these criteria we classified the trophic status of the rivers studied, from upstream to downstream in the CRW, as ranging from oligotrophic, through mesotrophic to eutrophic, and some downstream parts were even classified as hypereutrophic. These results show that there are strong anthropogenic interferences (industrial and agricultural activities, urbanization) in the rivers in the CRW, and these interferences have caused the river water quality to deteriorate severely.

**Table 1:** TN, TP and heavy metal concentrations in the water from the studied rivers in the Chaobai River Watershed (each concentration is the mean ± the standard deviation).

Site	TN	TP	Cr	Ni	Cu	Pb
	mg/L	µg/L	µg/L	µg/L	µg/L	µg/L
Bai River and its tributaries	2.4±1.4	17±11	1.2±1.1	1.2±1.1	3.2±4.1	1.0±1.0
Chaobai River and its tributaries	6.5±4.3	608±952	6.8±7.9	6.3±4.2	21±63	1.3±0.8

### Effect of Eutrophication Conditions (N and P Concentrations) on the Periphyton Community

We found great morphological differences in the periphyton in the HRB, indicating that there were wide variations in the periphyton communities in different areas. Algae are the main contributors to the periphyton, and their conformations differ greatly, from unicellular diatoms to filamentous algae. Many methods for classifying periphyton types have been published previously. For example, Maltalis and Vincent divided periphyton into black crust, brown film, green crust, and green filaments by their colors and other characteristics [28]. The method used in a particular study is generally selected based on the specific requirements of the research. In our study, classifying the periphyton by morphology will allow us to identify eutrophication conditions (high N and P concentrations), and help to assess the water quality.

Periphyton transitions from unicellular diatoms to filamentous algae were observed along the river flow direction in the CRW, and these transitions were consistent with the observed changes in trophic status. The periphyton was divided into three different types, based on their conformations (Table 2). Type A was primarily epilithic unicellular diatoms, such as *Fragilaria* and *Cymbella*. Type B was filamentous algae with epiphytic diatoms (a hybrid type). Typical type B periphyton was *Cladophora* with *Tabellaria* attached. Type C was filamentous algae, such as *Cladophora* and *Oscillatoria*, with few (a biomass <5% of the total) or no unicellular diatoms.

**Table 2:** Main species found in the three periphyton types found in the rivers in the Chaobai River Watershed and the eutrophication conditions (TN and TP concentrations) in the ambient water.

	Type A	Type B	Type C
	*Fragilaria*	*Cladophora+Tabellaria*	*Cladophora*
	*Cymbella*	*Cladophora+Cocconeis*	*Oscillatoria*
	*Navicula*	*Cladophora+Cocconeis+Tabellaria*	*Spirogyra*
Main species	*Gomphonema*	*Cladophora+Tabellaria+Fragilaria*	*Ulothrix*
	*Cocconeis*	*Cladophora+Tabellaria+Gomphonema*	*Oedogonium*
		*Ulothrix+Cocconeis+Fragilaria*	
		*Oedogonium+Cocconeis+Fragilaria*	
		*Spirogyra+Navicula +Fragilaria+Cymbella*	
TN concentration (mg/L)	1.1±0.36	1.8±1.1	4.9±1.8
TP concentration (µg/L)	12±4.1	16±15	292±440

Many factors affect the periphyton community, including light, temperature and availability of nutrients in the water [24]. We selected sampling sites with similar physical and hydrological conditions so that the effects of eutrophication (high N and P concentrations) could be identified. Areas that had type B periphyton communities had slightly higher TN and TP concentrations in the river water than the areas with type A periphyton communities (Table 2), but areas with type C periphyton communities had much higher TN and TP concentrations in the water. We concluded that increasing nutrient concentrations travelling downstream were the main factors causing the changes in the periphyton communities, and this agrees with the results of a study conducted by Kelly [29]. As has been shown previously, seasonal variations in the concentrations of some metals in river periphyton can be related to variations in the periphytic algae and cyanobacteria species present [30]. Therefore, we believe that the changes in the periphytic assemblages we found may have been connected with heavy metal accumulation by the periphyton.

### Heavy Metal Accumulation by Different Periphyton Types

**Figure 2:**
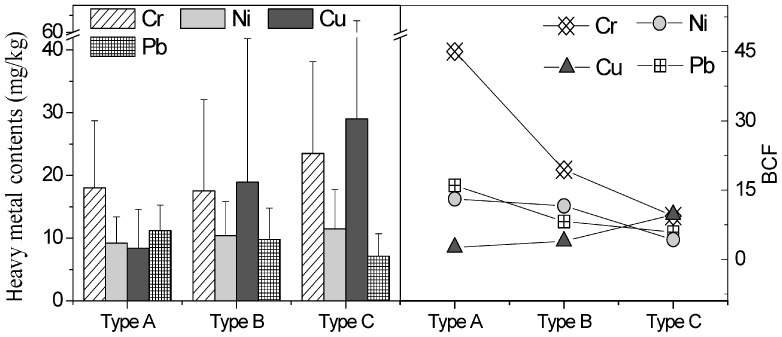
Heavy metal concentrations in the three types of periphyton and their bioconcentration factors (BCFs).

There were great variations in the amounts of the heavy metals accumulated by the different periphyton types (Fig. 2). The Cr concentrations were 18±11 and 18±15 mg/kg in periphyton types A and B, respectively, but 24±15 mg/kg in type C. Cr was enriched in the periphyton in the decreasing order type A (BCF = 45)>type B (BCF = 19)>type C (BCF = 9.4). The Ni concentrations in the three periphyton types were not significantly different (type A 9.2±4.2 mg/kg, type B 10±5.4 mg/kg, type C 12±6.2 mg/kg), even though the Ni concentration in the ambient water increased moving downstream (Table 1). The Ni BCF values, therefore, decreased in the order type A (13)>type B (12)>type C (4.3). The excretion of extracellular organic matter by diatoms enables them to scavenge metals, and may provide the diatoms with more resistance to Ni toxicity [31]. The high Ni BCF for type A (unicellular diatoms) suggested that they have a strong ability to accumulate Ni, which is consistent with previous findings. The Pb BCF values (type A 16, type B 8.2, type C 5.9) explained the decrease in Pb concentration moving from type A (11±4.1 mg/kg), through type B (9.8±5.0 mg/kg), to type C (7.1±3.6 mg/kg).

Unlike the Cr, Ni and Pb concentrations, the Cu concentrations increased clearly in the order type A (8.4±6.2 mg/kg)<type B (19±23 mg/kg)<type C (29±35 mg/kg). The Cu BCF values also increased in the order type A (2.6)<type B (3.9)<type C (9.7). These results indicate that the filamentous algae (type C) sampled could accumulate more Cu than the unicellular diatoms (type A) could. The presence of Cu could influence the rate of photosynthesis in the diatoms more than in the filamentous algae [32]. In our study, the filamentous algae gradually became the dominant species moving from type A to type C, so type C would be the most resistant to the effects of Cu, and this may have caused the stronger ability of type C to enrich Cu.

Overall, the abilities of the periphyton to accumulate Cr, Ni and Pb decreased but the ability to accumulate Cu increased moving from type A to type C in the CRW rivers. Previous studies have shown that filamentous algae, such as *Cladophora*, are generally the best bioindicators for heavy metals in aquatic ecosystems [25]. In our study, the changes in periphyton types found (from type A to type C) caused a significant increase in the ecological risk associated with Cu pollution, but a decrease in the ecological risks associated with Cr, Ni and Pb pollution.

### Correlation Analysis of the Heavy Metal Concentrations in Periphyton

**Figure 3:**
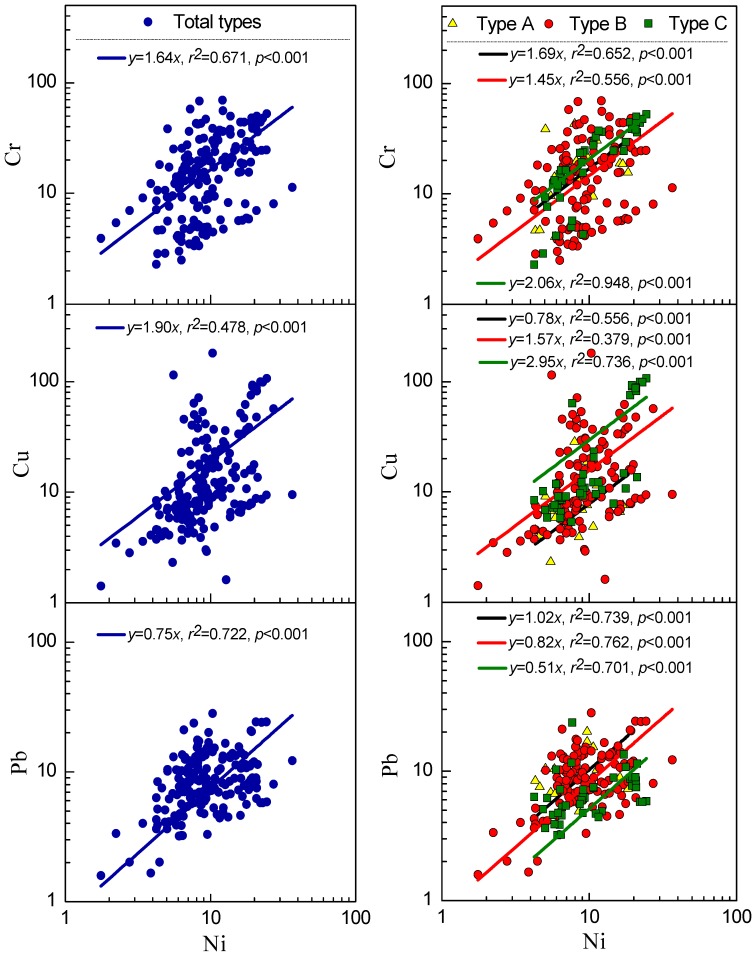
Linear regressions between the Ni concentration and the Cr, Cu and Pb concentrations (mg/kg) in the three periphyton types (as a total on the left and separately on the right). The concentrations were log-transformed.

The relationships between the heavy metal concentrations in the three periphyton types were analyzed using Spearman’s correlations (Table 3). Strong positive correlations (p≤0.01) were found between the Cr and Cu concentrations, the Cr and Pb concentrations, and the Cu and Pb concentrations in type A periphyton. Except for between the Cr and Cu concentrations in type B and the Cr and Pb concentrations in type C, all four heavy metals positively correlated with each other in periphyton types B and C. There was a considerable increase in the significance of the correlations between Ni and the other three metals (Cr, Cu and Pb) moving from periphyton types A through to C. Therefore, we produced scatter plots of the Ni concentration and the concentrations of the other three metals, for each of the periphyton types, and fitted a line to each (Fig. 3). There tend to be correlations between the accumulations of different heavy metals in periphyton [33], [34]. In our study, the Cr, Cu and Pb concentrations all increased with the Ni concentration in all three periphyton types. The Cr:Ni ratios decreased in the order type C>type A ≈ type B and the Cu:Ni ratios decreased in the order type C>type B>type A. However, the Pb:Ni ratios gradually decreased moving from type A through to type C. The Ni concentration was a good predictor of the Cr, Cu and Pb accumulation in periphyton types A, B and C, and the differences in Ni (and Cr, Cu and Pb) accumulation between the filamentous algae and unicellular diatoms may be caused by different co-accumulation effects. Periphyton type A had a different ability to accumulate heavy metals from type C (Figs. 2 and 3). The increasing TN and TP concentrations resulted in the changes seen in the periphyton community types in the studied rivers. Therefore, we concluded that eutrophication (increased N and P concentrations) were important factors affecting the accumulation of heavy metals by periphyton in the rivers in the CRW.

**Table 3:** Spearman correlation coefficients for the heavy metal concentrations in the periphyton.

	Type	Cr	Ni	Cu	Pb
**Cr**	**A**	1	0.278	0.863^a^	0.595^a^
	**B**	1	0.341^a^	−0.184^b^	0.365^a^
	**C**	1	0.904^a^	0.711^a^	0.188
**Ni**	**A**		1	0.331	0.251
	**B**		1	0.453^a^	0.371^a^
	**C**		1	0.799^a^	0.466^a^
**Cu**	**A**			1	0.598^a^
	**B**			1	0.194^b^
	**C**			1	0.476^a^
**Pb**	**A**				1
	**B**				1
	**C**				1

a Correlation is significant at the 0.01 level (2-tailed).

b Correlation is significant at the 0.05 level (2-tailed).

## Conclusions

Changes in the periphyton assemblages, from unicellular diatoms to filamentous algae, were observed moving along the flow direction in the rivers studied, and these changes were consistent with changes in the trophic status. The periphyton were classified into type A (unicellular diatoms), type B (filamentous algae with diatoms), and type C (filamentous algae), based on their morphologies. The changes in periphyton types caused a significant increase in the ecological risk associated with Cu pollution but decreased risks associated with Cr, Ni and Pb pollution. The Ni concentration was a good indicator of the Cr, Cu and Pb accumulation in the periphyton. These results provide information that will be useful for designing further research and for managing the rivers in the study area, and will help enable heavy metal pollution controls to be established in the HRB.
